# Melanocortin 2 receptor antagonists in canine pituitary-dependent hypercortisolism: in vitro studies

**DOI:** 10.1007/s11259-018-9737-x

**Published:** 2018-09-05

**Authors:** Karin Sanders, Jan A. Mol, Hans S. Kooistra, Sara Galac

**Affiliations:** Department of Clinical Sciences of Companion Animals, Faculty of Veterinary Medicine, Utrecht University, Yalelaan 108, 3584 CM Utrecht, the Netherlands

**Keywords:** Cushing’s syndrome, PDH, Treatment, ACTH, MC2R, Dog

## Abstract

Canine hypercortisolism is most often caused by an ACTH-secreting pituitary adenoma (pituitary-dependent hypercortisolism; PDH). An interesting target for a selective medical treatment of PDH would be the receptor for ACTH: the melanocortin 2 receptor (MC2R). In this study we investigated whether two peptide compounds, BIM-22776 (#776) and BIM-22A299 (#299), are effective MC2R antagonists in vitro. Their effects on cortisol production and mRNA expression of steroidogenic enzymes, MC2R and melanocortin 2 receptor accessory protein (MRAP) were evaluated in primary adrenocortical cell cultures (*n* = 8) of normal canine adrenal glands. Cortisol production stimulated by 50 nM ACTH was dose-dependently inhibited by #299 (inhibition 90.7 ± 2.3% at 5 μM) and by #776 (inhibition 38.0 ± 5.2% at 5 μM). The ACTH-stimulated mRNA expression of steroidogenic enzymes, MC2R and MRAP was significantly inhibited by both compounds, but most potently by #299. These results indicate that canine primary cell culture is a valuable in vitro system to test MC2R antagonists, and that these compounds, but especially #299, are effective MC2R antagonists in vitro. To determine its efficacy in vivo, further studies are warranted. Antagonism of the MC2R is a promising potential treatment approach in canine PDH.

## Introduction

Hypercortisolism (Cushing’s syndrome) is one of the most frequently diagnosed endocrinopathies in dogs (Galac et al. 2010c). This serious endocrine disorder is characterized by chronic exposure to excessive amounts of glucocorticoids, which can be caused by either glucocorticoid administration or endogenous cortisol overproduction. Endogenous hypercortisolism occurs in approximately 1 to 2.5 per 1000 dogs per year (Willeberg and Priester 1982; O’Neill et al. 2016), and is most frequently (~80–85%) caused by an ACTH-producing pituitary adenoma (pituitary-dependent hypercortisolism; PDH) (Galac et al. 2010c).

The current drug of choice for the medical treatment of canine PDH is trilostane, which competitively inhibits the steroidogenic enzyme 3β-hydroxysteroid dehydrogenase type 2 (HSD3B2) (Potts et al. 1978; Ramsey 2010). However, HSD3B2 is required for all classes of adrenocortical hormones and therefore not only inhibits the production of cortisol but also that of aldosterone (Galac et al. 2010a; Reid et al. 2014). Although trilostane is generally well tolerated, hypoadrenocorticism can occur (King and Morton 2017), and adrenal necrosis might occur more commonly than generally thought (Reusch et al. 2007), possibly due to increased ACTH secretion (Burkhardt et al. 2011). A more selective treatment option where the negative effects of increased ACTH secretion are countered could therefore improve the current medical treatment of canine PDH.

An interesting target for a more selective medical treatment of PDH would be the receptor for ACTH: the G_sα_-protein-coupled melanocortin 2 receptor (MC2R) (Mountjoy et al. 1992). The MC2R is expressed in all zones of the adrenal cortex, but its major function is to stimulate the zona fasciculata cells to produce cortisol (Clark et al. 2016; Sanders et al. 2016). The MC2R is one of five melanocortin receptors: MC1R-MC5R, which are all activated by melanocortin peptides that are derived from the precursor pro-opiomelanocortin (Bicknell 2008). The MC2R is unique in its ligand selectivity: while multiple melanocortin peptides can bind to the other MC receptors, only ACTH binds to the MC2R (Cerdá-Reverter et al. 2012; Dores et al. 2014). The MC2R needs to be transported from the endoplasmic reticulum to the cell surface, for which it requires the melanocortin 2 receptor accessory protein (MRAP). MRAP forms a complex with the MC2R which allows the MC2R to leave the endoplasmic reticulum and reach the cell surface, and which is necessary for binding of ACTH to the MC2R (Cooray et al. 2011; Cerdá-Reverter et al. 2012). This binding activates the cAMP-protein kinase A pathway which facilitates cholesterol transport to the inner mitochondrial membrane and the phosphorylation of several transcription factors. These activated transcription factors then increase the availability of free cholesterol and the transcription of genes encoding for steroidogenic enzymes, which eventually results in increased cortisol production (Stocco 1997; Ruggiero and Lalli 2016).

Consequently, a potent MC2R antagonist would be a great new treatment option to selectively inhibit ACTH-dependent hypercortisolism. The aim of this study was to evaluate whether the two peptide compounds BIM-22776 (#776) and BIM-22A299 (#299) are potent MC2R antagonists in vitro, and to determine whether MC2R antagonists have potential as a future treatment option for canine PDH.

## Materials & methods

### Animals & tissues

The adrenal glands of eight healthy dogs were used. The dogs were euthanized for reasons unrelated to this study, which was approved by the Ethical Committee of Utrecht University. The dogs were between 18 and 48 months of age (median 23 months), two were mongrels and six were beagles. One dog was female and seven were male, all of the dogs were sexually intact.

### Primary cell culture

The adrenocortical cell suspensions were prepared as described previously (Sanders et al. 2018). In short, the adrenal cortices were digested in a collagenase solution, then filtered and washed. The cell suspensions were diluted to 1 × 10^5^ cells/mL with DMEM F-12 (Gibco, Invitrogen, Breda, the Netherlands) with 1% Insulin-Transferrin-Selenium (Gibco), 0.125% BSA, 2.5% Nu-Serum (Corning, Amsterdam, the Netherlands) and 1% penicillin/streptomycin, and seeded in Multiwell 96 well plates (100 μL per well) (Primaria™, Corning).

Two 96 well plates were used for each adrenal cell suspension: one plate for cortisol/DNA ratio measurements and one plate for reverse transcription quantitative PCR (RT-qPCR) analysis. The cells were left to attach for 4 to 7 days at 37 °C in a humidified atmosphere of 95% air and 5% CO_2_, after which the culture medium was refreshed prior to compound incubations.

Stock solutions of 500 μM were prepared for compounds #776 and #299, dissolved in 10 mM HCl. The cells were incubated with 50 nM ACTH (1–24) (Synacthen®, Sigma-tau BV, Utrecht, the Netherlands) and 50 nM, 500 nM and 5 μM of #776 and #299. To determine whether the compounds would only affect cortisol production when ACTH(1–24) was added, cells were also incubated without ACTH(1–24) and with 5 μM of #776 and #299. Incubations were performed in quadruplicate. After 24 h of incubation, cortisol concentrations were measured in the culture medium of four wells per condition by radioimmunoassay as described previously (Meijer et al. 1978).

### DNA measurements

To correct for differences in number of cells per well, DNA was measured in each well to calculate cortisol/DNA ratios. After removing the culture medium, the culture plates underwent three freeze/thaw cycles, after which 50 μL Tris/EDTA (10 mM Tris, 1 mM EDTA, pH 8.0) was added to each well. The Qubit® dsDNA HS Assay Kit (Fisher Scientific, Landsmeer, the Netherlands) was used according to the manufacturer’s instructions and DNA concentrations were measured with the Qubit® 2.0 Fluorometer (Fisher Scientific). The cortisol/DNA ratios were calculated of four wells per condition, of which the results were averaged prior to statistical analysis.

### RT-qPCR

After removing the culture medium, the wells for each condition were pooled and RNA was isolated from the cells with the RNeasy Micro Kit (QIAGEN, Venlo, the Netherlands), including the DNase treatment, according to the manufacturer’s instructions. RNA concentrations were measured with NanoDrop (ND-1000, Isogen Life Science, Utrecht, the Netherlands), after which cDNA was synthesized from 500 ng total RNA with the iScript™ cDNA Synthesis Kit (Bio-Rad) according to protocol. The cDNA was subsequently diluted to 1 ng/μL. RT-qPCR analysis was used to determine the mRNA expression of eight genes: steroidogenic acute regulatory protein (StAR), cytochrome P450 side chain cleavage (CYP11A1), HSD3B2, 17a-hydroxylase/17,20-lyase (CYP17), 21-hydroxylase (CYP21), 11β-hydroxylase cytochrome P450 (CYP11B1), MC2R and MRAP (primers shown in Table 1). Optimization and confirmation of the primer specificity were performed as described previously (Galac et al. 2010b).

**Table 1:** Primers used for RT-qPCR

Target gene	Primer sequence (5′ → 3′)	Product size (bp)	Annealing Tm (°C)
StAR	Fw: CTC TGC TTG GTT CTC GG Rv: CCT TCT TCC AGC CTT CC	125	62.5
CYP11A1	Fw: CAC CGC CTC CTT AAA AAG TAA CAA G Rv: GCT GCG TGC CAT CTC GTA G	129	63.3
CYP17	Fw: CCT GCG GCC CCT ATG CTC Rv: GGC CGG TAC CAC TCC TTC TCA	134	60.0
HSD3B2	Fw: CAG GAG GGT TTC TGG GTC AG Rv: AGG CTC TCT TCA GGC ACT GC	186	56.5
CYP21	Fw: AGC CCG ACC TTC CCC TCC ACC TG Rv: TCT GCC GGC GAA GTC CAC CCA TTT	152	64.5
CYP11B1	Fw: GCC TAC CCC TTG TGG ATG AC Rv: CTC TGT GAC TGC TGT CTG GG	126	62.0
MC2R	Fw: TCA TGT GGT TTT GCC GGA AGA GAT Rv: AAT GGC CAG GCT GCA AAT GAA A	138	58.5
MRAP	Fw: CAC AGG TGA GGA ACA ACG Rv: ATC GAA GGT CAG TCC TGG	227	64.6
RPS19	Fw: CCT TCC TCA AAA AGT CTG GG Rv: GTT CTC ATC GTA GGG AGC AAG	95	61.0
SDHA	Fw: GCC TTG GAT CTC TTG ATG GA Rv: TTC TTG GCT CTT ATG CGA TG	92	61.0
HPRT	Fw: AGC TTG CTG GRG AAA AGG AC Rv: TTA TAG TCA AGG GCA TAT CC	104	58.0
YWHAZ	Fw: CGA AGT TGC TGC TGG TGA Rv: TTG CAT TTC CTT TTT GCT GA	94	58.0

To correct for differences in cDNA concentrations, Ribosomal protein S19 (RPS19), succinate dehydrogenase complex subunit A (SDHA), hypoxanthine-guanine phosphoribosyltransferase (HPRT) and tyrosine 3-monooxygenase/tryptophan 5-monooxygenase activation protein zeta (YWHAZ) were used as reference genes (primers shown in Table 1) (Brinkhof et al. 2006; Stassen et al. 2015).

SYBRgreen supermix (Bio-Rad) was used for the RT-qPCR reactions, and amplification was performed using a CFX 384 Touch™ Real-Time PCR Detection System (Bio-Rad) with the following cycle parameters: initial denaturation for 3 min at 95 °C, then 40 cycles of 10 s at 95 °C followed by 30 s at the primer-specific optimal annealing temperature, and ending with melting curve analysis by one cycle of 10 s 95 °C and a temperature increment of 0.5 °C for 5 s from 65 °C to 95 °C. To exclude the possibility of interfering genomic DNA, for each sample a control where no reverse transcriptase was added in the cDNA reaction was analyzed. Data were analyzed with CFX Manager 3.1 (Bio-Rad). Two technical replicates were used for each sample. GeNorm software (Vandesompele et al. 2002) was used to analyze expression levels of the reference genes, which justified their use. To calculate the normalized relative expression of each target gene, the 2^-ΔΔCt^ method (Livak and Schmittgen 2001) was used.

### Statistical analysis

Logarithmic transformation resulted in normally distributed data, which was confirmed with the Shapiro-Wilk test. After logarithmic transformation, cortisol/DNA ratios and RT-qPCR fold changes were analyzed with repeated measures ANOVA with a Bonferroni post-hoc test to correct for multiple comparisons. A *p* value of <0.05 was considered significant. Data are reported as mean ± SEM of eight individual cell cultures.

## Results

### Cortisol production: cortisol/DNA ratios

**Fig. 1:**
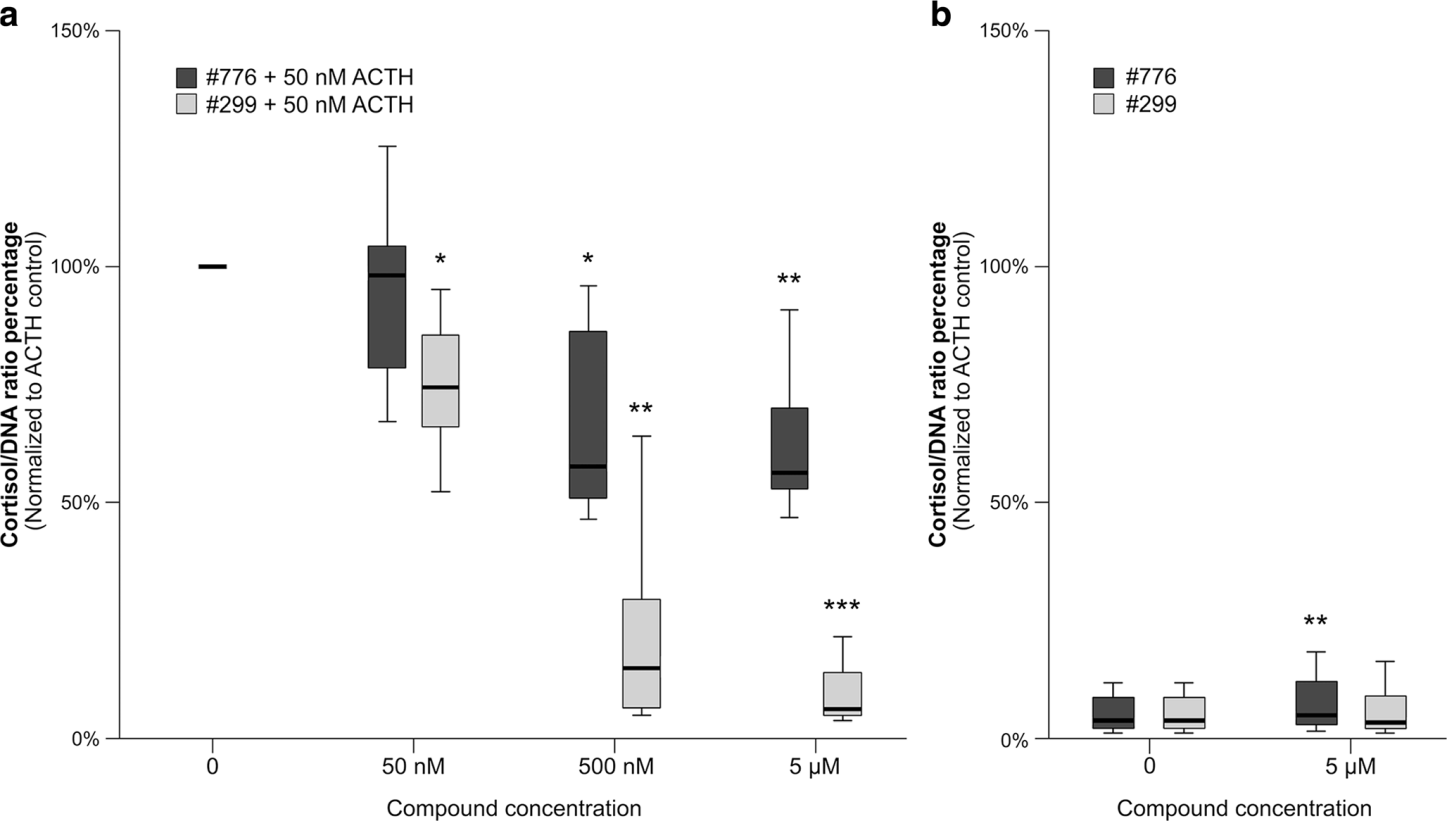
The effects of compounds BIM-22776 (#776) and BIM-22A299 (#299) on the cortisol production of ACTH(1–24)-stimulated (A) and non-ACTH-stimulated (B) canine primary adrenocortical cell cultures (*n* = 8). Cortisol/DNA ratios are shown in percentages, normalized to the ACTH-stimulated control. Asterisks represent significant differences compared to the ACTH-stimulated controls: *P < 0.05, **P < 0.01, ***P < 0.001

Incubation with 50 nM ACTH(1–24) increased the cortisol/DNA ratio 35.4 ± 10.4-fold (*p* < 0.0001). Co-incubation with #776 dose-dependently inhibited the ACTH-stimulated cortisol/DNA ratio by 33.5 ± 7.1% at 500 nM and by 38.0 ± 5.2% at 5 μM (Fig. 1A). Co-incubation with #299 dose-dependently inhibited the ACTH-stimulated cortisol/DNA ratio by 25.1 ± 5.0% at 50 nM, by 78.8 ± 7.2% at 500 nM and by 90.7 ± 2.3% at 5 μM (Fig. 1A).

In the non-ACTH-stimulated cells, neither compound inhibited the cortisol/DNA ratio. On the contrary, #776 slightly but significantly (*p* = 0.002) increased the non-ACTH-stimulated cortisol/DNA ratio 1.4 ± 0.1-fold at 5 μM (Fig. 1B). Compound #299 did not affect the non-ACTH-stimulated cortisol/DNA ratio (1.1 ± 0.1-fold, *P* = 1) (Fig. 1B).

### RT-qPCR

**Fig. 2:**
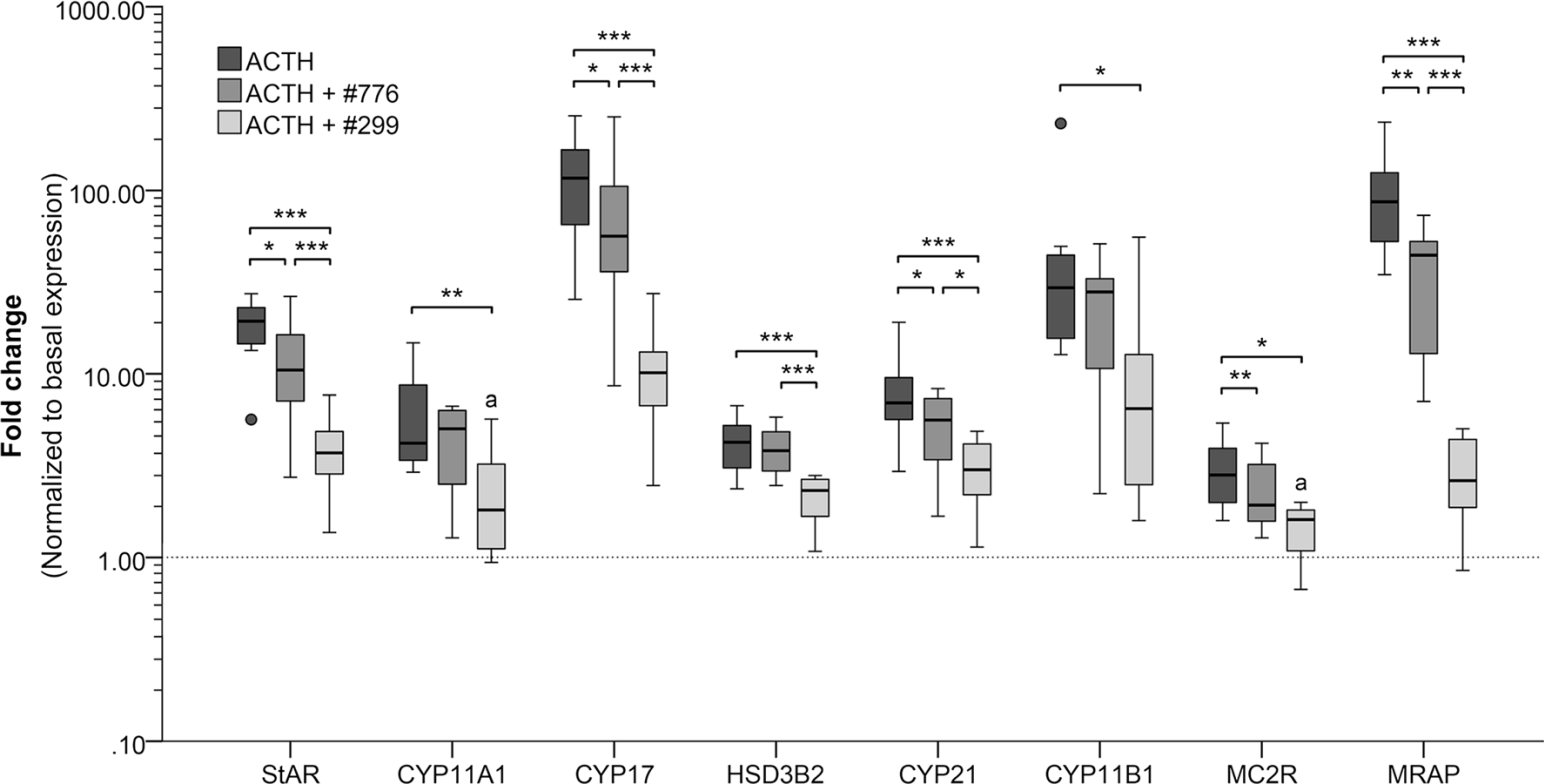
The effects of incubation with 50 nM ACTH(1–24) and of co-incubation of ACTH(1–24) with 5 μM of compounds BIM-22776 (#776) and BIM-22A299 (#299) on the relative mRNA expression of steroidogenic enzymes, MC2R, and MRAP in canine primary adrenocortical cell cultures (n = 8). Fold changes are normalized to the non-ACTH-stimulated controls, i.e. the basal expression. Asterisks represent significant differences: *P < 0.05, **P < 0.01, ***P < 0.001. All conditions were significantly different from the basal expression, except when indicated with an “a”. StAR, steroidogenic acute regulatory protein; CYP11A1, cytochrome P450 side chain cleavage; CYP17, 17α-hydroxylase/17,20-lyase; HSD3B2, 3β-hydroxysteroid hydrogenase type 2; CYP21, 21-hydroxylase; CYP11B1, 11β-hydroxylase cytochrome P450; MC2R, melanocortin 2 receptor; MRAP, melanocortin type 2 accessory protein

Incubation with 50 nM ACTH(1–24) significantly (*P* < 0.01 or lower) upregulated the mRNA expression of all the genes analyzed in this study, but most notably that of CYP17, followed by MRAP, CYP11B1 and StAR (Fig. 2). Co-incubation with 5 μM #776 significantly inhibited the ACTH-stimulated expression of five of the eight genes analyzed in this study (Fig. 2), while co-incubation with 5 μM #299 significantly inhibited the ACTH-stimulated expression of all the genes analyzed in this study (Fig. 2).

## Discussion

The results of this study show that canine primary adrenocortical cell culture stimulated with synthetic ACTH(1–24) is a functional in vitro model to test the efficacy of MC2R antagonists. Moreover, this study shows that #299 and #776 are effective MC2R antagonists, of which #299 is the most potent.

Multiple attempts to create or isolate MC2R antagonists have been made previously (Seelig and Sayers 1973; Yang et al. 1997; Dores 2013), mostly with varying effects. Recently, Bouw et al. (2014) showed that GPS1573 and GPS1574, two ACTH analogs, can antagonize MC2R in vitro in the nanomolar range in a human embryonic kidney cell line transfected with the MC2R (Bouw et al. 2014). However, a subsequent study by Nensey et al. (2016) demonstrated that GPS1573 could not antagonize the adrenal response to ACTH in neonatal rats in vivo. High concentrations of GPS1574 did dose-dependently inhibit corticosterone production in these rats (Nensey et al. 2016). Whether #776 and #299 can antagonize the adrenal response to ACTH in vivo remains to be determined, but using primary adrenocortical cell cultures might be a better predictor of in vivo functionality than using homogeneous and genetically altered cell lines from extra-adrenal sources.

In this study we evaluated how the compounds affected the cortisol production of both ACTH-stimulated and non-ACTH-stimulated cells. We aimed to mimic ACTH-dependent hypercortisolism by adding 50 nM synthetic ACTH(1–24). This ACTH concentration significantly and strongly increased the cortisol production, which indicates that the cells responded as expected and that canine primary adrenocortical cell culture is a good in vitro model to test the effects of ACTH. Because we corrected the cortisol values with the DNA concentrations, we could exclude the possibility that any observed differences in the cortisol production were caused by a difference in the number of cells.

In the non-ACTH-stimulated canine adrenocortical cells, incubation with #776 slightly but significantly increased the cortisol production, which could indicate that #776 has agonistic properties when the natural agonist is absent. Since using MC2R antagonists in a clinical setting would only be indicated when ACTH is excessively secreted, this phenomenon is expected to be clinically irrelevant. Incubation with #299 did not affect non-ACTH-stimulated cortisol production.

To evaluate whether the compounds were able to antagonize the ACTH-induced changes in the mRNA expressions of steroidogenic enzymes, the MC2R and MRAP, we performed RT-qPCR analyses. ACTH upregulated the mRNA expressions of all the genes analyzed in this study, while #299 inhibited the ACTH-stimulated mRNA expressions of these genes. These results show that #299 can antagonize the ACTH-induced changes in the mRNA expressions of steroidogenic enzymes, the MC2R and MRAP. Co-incubation with #776 downregulated the ACTH-stimulated mRNA expression of most of the genes analyzed in this study, but not of all genes and not as vigorously as #299.

One of the advantages of using MC2R antagonists is that functional MC2R expression is limited to the adrenal gland; antagonism of the MC2R is therefore unlikely to result in off-target effects. However, since the other melanocortin receptors have a variety of functions in other tissue types, inadvertently antagonizing or agonizing these receptors could result in many unwanted side-effects (Clark et al. 2016). It is therefore important to determine whether the compounds are selective for the MC2R, and do not affect the other melanocortin receptors. This compound selectivity will have to be determined in future studies.

## Conclusion

In conclusion, the results of this study indicate that canine primary cell culture is a valuable in vitro system to test MC2R antagonists, and that these compounds, but especially #299, are effective MC2R antagonists in vitro. To determine their efficacy in vivo, further studies are warranted. Antagonism of the MC2R is a promising potential treatment approach in canine PDH.
